# Relation of injuries and psychological symptoms in amateur soccer players

**DOI:** 10.1136/bmjsem-2019-000522

**Published:** 2019-04-24

**Authors:** Petra Jansen, Jennifer Lehmann, Birgit Fellner, Gunnar Huppertz, Oliver Loose, Leonard Achenbach, Werner Krutsch

**Affiliations:** 1 Faculty of Psychology, Pedagogy and Sports Science, University of Regensburg, Regensburg, Bavaria, Germany; 2 Centerof Clinical Studies, University Medical Centre Regensburg, Regensburg, Bavaria, Germany; 3 Clinic of Pediatric Surgery, Clinic St. Hedwig, Regensburg, Bavaria, Germany; 4 Department of Trauma Surgery, University Medical Centre Regensburg, Regensburg, Bavaria, Germany

**Keywords:** injury, depression, anxiety, self-compassion, amateur soccer players

## Abstract

**Objectives:**

The first main goal of this study was to investigate the prevalence of depression and anxiety as well as self-compassion in a heterogeneous sample of male amateur soccer players. The second main goal of this study was the examination of the relationship between injuries and psychological factors in amateur soccer players.

**Methods:**

Players were recruited from German amateur soccer clubs of the fourth to seventh league. 419 soccer players with the mean age of 22.88 years participated in the psychological and the injury assessment at the beginning of the season and at the end, 9 months later. For the psychological assessment, depression and anxiety rate as well as self-compassion was analysed. Furthermore, the frequencies of injuries were registered.

**Results:**

The results showed that players of the highest amateur league, the fourth league in German soccer, showed significantly higher anxiety values than players from a lower league (p=0.013). There were no differences in depression values dependent on the league. Furthermore, players who suffered from an injury before the start of the season demonstrated higher anxiety values (p=0.027). This result was independent of the respective league.

**Conclusion:**

The results of this study demonstrate that even in higher amateur soccer the anxiety level of the players varies between soccer players of different leagues. Because an injury before the start of the season influenced the anxiety level, a psychological treatment during injury should be considered.

**Summary box**

- Even in higher amateur soccer the anxiety level of the players varies between soccer players of different leagues.
- Psychological factors are also in lower soccer levels related to injuries.
- The possible relation of psychological and medical issues must be also investigated in amateur soccer players.

## Introduction

Soccer is one of the most famous sports in the world. Up to 260 million people all over the world are registered in soccer clubs, with several levels ranging from professional to amateur soccer.^1^ Even though soccer is very popular, it is also accompanied by a high risk of injuries. The prevalence of at least one injury in one season in the semiprofessional German salaried soccer leagues was 79%.^2^ Besides the topic of injury risk and injury prevention in soccer, the possible relevance of mental health problems, that means emotional or psychological, gets more and more prominent because of the tragic suicide of famous soccer players and because of the statements of national players that the high pressure in professional soccer leads to symptoms of anxiety and depression.

### Psychological symptoms in soccer

Until now, all relevant studies concerning possible psychological symptoms in soccer were conducted in the area of elite soccer. Thereby, the mental or psychological health issue has two sides: on the one side sports can prevent mental health disorders,^3^ on the other side the high pressure in professional soccer might contribute to a possible depression or anxiety development.^4^ There are several investigations with mostly professional athletes examining the topic of depression in sports, especially in soccer. In one study with professional soccer players, Junge and Feddermann-Demont^5^ investigated the appearance of depression and anxiety in players of all first leagues and of four U-21 soccer teams in Switzerland.^5^ Their results showed that the prevalence of depression was similar to the general population (7.6% showed a mild to moderate depression and 3% a major depression) and the prevalence of anxiety was significantly lower (16.7%). However, the results showed that male U-21 professional players had a higher prevalence of depression. In this study, a highly selected sample was chosen, that means a highly homogenous and representative group of top-level male and female athletes. In contrast to this study, Gouttebarge *et al*^6^ demonstrated that a high prevalence of common mental disorders (CMD) like depression and anxiety and adverse health behaviour like adverse alcohol consumption, smoking and unhealthy nutrition behaviour could be detected among active professional soccer players, who had to be a member of national player’s union.^6^ Gouttebarge *et al*^7^ demonstrated further that the number of severe musculoskeletal injuries during a soccer career was positively correlated with distress, anxiety and sleeping disturbance. In addition, the number of surgeries was correlated with adverse alcohol behaviour.^7^ This is in line with a study of Kiliç *et al*^8^, who found that anxiety and depression show the highest prevalence of all relevant aspects of CMDs in active or retired professional soccer players (18% and 19%).^9^ Their participants play or played soccer at the highest professional soccer leagues. Kiliç *et al*^8^ demonstrated also that on the one hand there was no relationship between symptoms of CMD and the onset of severe musculoskeletal timeless injuries, but on the other hand soccer players who suffer from severe musculoskeletal time-loss injuries are likely to develop subsequent symptoms of CMD. This is in accordance with the fact that former players with depressive symptoms mentioned more often injuries as a reason to retire.^10^ Until now, the existence of prospective studies regarding the topic of mental health in soccer is rare and, furthermore, there is no study which investigates this topic in non-elite soccer players.

### Aims of the study

There are three main aims of this study:

1. First, we want to investigate the prevalence of depression and anxiety as well as self-compassion in a heterogeneous sample of male amateur soccer players. Until now there were only studies in elite soccer players and the results contradict in some way. Whereas Junge and Feddermann-Demont [5] found that the depression rate of elite soccer players resembles the one of the general population who do not play soccer as professionals, Gouttebarge *et al* [6] showed a higher prevalence of mental health complaints, like depression. According to those results, it has to be investigated if the depression and anxiety rate of amateur soccer players is similar, lower or higher as in the non-soccer-playing population.
2. Additionally, it is the goal of this study to focus on the negative emotional aspects of soccer and on the positive ones. Therefore, we also investigate the appearance of self-compassion in soccer players. Self-compassion is the ability to hold one’s feelings of suffering with a sense of warmth, connection and concern.^11^ Self-compassion is an important protective factor, fostering emotional resilience.^12^ It has been demonstrated that a self-compassion intervention is a potential coping resource for women athletes^13^ but has never been investigated with soccer players so far.
3. Third, we would like to investigate the relationship of injuries and emotional states in amateur soccer players. Until now, there were no studies, which have investigated this topic in more detail. According to the study of Kiliç *et al*^8^ in professional soccer players, we assume that injuries lead to negative emotional states, but that negative emotional states do not lead to more injuries during soccer season.

## Methods

The study used a quasiexperimental (correlational) design. It was part of a large national interventional research project to analyse and improve injury prevention in German amateur soccer players. In this interventional study screening tests were established to identify the risk factors for injuries. One part of those screening tests was the application of psychological tests. After the screening tests, a prevention intervention programme was applied.^2^ The study was conducted in collaboration with the Regional Football Association Bayern (1.6 million players, largest regional Football Association under the German Football Association) and the Public Trauma Insurance for elite salaried athletes in Germany, Verwaltungsberufsgenossenschaft.

### Participants

The leagues of this study population in amateur soccer range from the fourth to the seventh league of the Bavarian Football System and the highest junior levels under the age of 19. This amateur level includes in Germany salaried players in the highest amateur leagues and with main jobs outside of football, with common public sports trauma insurance, with more than three until four training sessions per week and with team coaches with similar soccer coach licence. According to Gouttebarge *et al*^6^ sample size calculation indicated that at least 138 participants were needed (power of 80%; 95% CI, precision of 5%) according to the assumption that 10% of players suffer from a mental negative health condition. This assumption is due to the study of Junge and Feddermann-Demont^5^ that between 7.6% and 16.7% of soccer players showed a mild depression or an anxiety disorder. In this study, 419 male soccer players from the four different semiprofessional leagues took part. The mean age was 22.88 (SD=4.02).

### Measurements

Participants completed a depression measurement (General Depression Scale, Allgemeine Depressions-Skala, ADS-L), an anxiety measurement (State-Trait Anxiety Inventory (STAI) trait) and a scale of self-compassion (Self-Compassion Scale, SCS).

#### Depression measurement (ADS-L)

For the depressive measurement, the General Depression Scale^14^ which measures the different stages of depressive symptoms was used. Through questioning the depressive symptoms of uncertainty, fatigue, hopelessness, demotion of oneself, dejection, loneliness, sadness, listlessness, fear, and so on, depression symptoms are analysed. Answers were corrected regarding their worthiness. Cronbach’s alpha was 0.81. A cut-off value of 23 or higher gives a hint for an existing depression.

#### Anxiety measurement (STAI trait)

Anxiety was measured with the *State-Trait Anxiety Test*.^15^ The State-Trait Anxiety Test measures trait and state anxiety, with 20 questions concerning state anxiety and 20 questions concerning trait anxiety. In this study, we only used the trait component. Cronbach’s alpha was between 0.88 and 0.94. A cut-off value of 46 or higher indicates an existing anxiety disorder.

#### Measurement of self-compassion (SCS)

The construct of self-compassion was examined with the SCS.^11^ This scale includes the following six elements of self-compassion: self-kindness, reduced self-judgement, common humanity, reduced isolation, mindfulness and reduced overidentification. Responses had to be given on a scale from 1 (almost never) up to 5 (almost always). The items self-kindness, common humanity and mindfulness were positively associated, and the items self-judgement, isolation and overidentification are negatively related. The overall score reached a Cronbach’s alpha=0.92 and the Cronbach’s alpha of the six subscales varied between 0.80 and 0.88.

### Procedure

All participants were explicitly informed about the study design and the psychological questionnaires by the authors in written form. At the pretest, 1527 soccer players answered the respective questionnaires and 419 completed the post-test, which resulted in a compliance rate of 27.4%. Pretest data were retrieved between March and August 2015 and post-test data between May and June 2016 in 62 different soccer clubs in Bavaria, Germany. At the beginning, psychological questionnaires were given as online version, which participants completed shortly (within 1 week) after the first meeting where they had given the respective information regarding their injuries. The frequencies of injuries in the following season were registered at the end of the season by a medical report, fulfilled by the team itself. The injury registration and injury assessment was based on the consensus statement of injury definition and data collection football.^16^ Informed consent was obtained from every participant.

### Statistical analyses

Continuous data are expressed as mean±SD and categorical data as frequency counts (percentages). For the analysis of the comparison of the depression, anxiety rate and self-compassion the data were set in relation to the percent range of the respective population. Furthermore, a univariate analysis of variance (ANOVA) was calculated for every dependent measurement with league as between-subject factor. The significance level was set to p<0.05, high significance to p<0.01. We repeated the above-mentioned ANOVAs with the factor group (players with an injury in the 3 months before the first testing, soccer players without). Besides, we correlated the frequency of injuries during the season with the psychological measurements. All analyses were performed using IBM SPSS Statistics, V.24.0.

## Results

All demographic and injury data are given in table 1.

**Table 1:** Descriptive data (reported as mean±SD, or percentage) and prevalence of symptoms of CMDs among soccer players in fourth to seventh league

Variables	Mean or N (%)	SD
Age (years)	22.88	±4.02
Height (cm)	181.29	±5.69
Weight (kg)	76.45	±7.04
Fourth league	N=57 (17.4%)	
Fifth league	N=109 (33.2%)	
Sixth league	N=125 (38.1%)	
Seventh league	N=37 (11.3%)	
Field position (n=325)		
Goalkeeper	N=36 (11.1%)	
Defender	N=149 (45.8%)	
Mid-fielder	nN=103 (31.7%)	
Forward	N=37 (11.4%)	
Injuries before the season		
General injuries in the last 3 months before the season (n=326)	N=117 (Yes: 35%)	
Knee injuries in the last 5 years before the season (n=322)	N=100 (Yes: 31%)	
Anterior cruciate ligament injury (n=322)	N=33 (10.2%)	
Posterior cruciate ligament injury (n=322)	N=3 (0.9%)	
Injuries during the season (n=366) Prevalence of psychological symptoms	N=156 (42.6%)	
Indices of a depression (critical value ≥23) (n=385)	nN=38 (9.8%)	
Indices of an anxiety disorder (critical value ≥45) (n=379)	N=73 (19.2%)	

CMD, common mental disorder.

*The psychological measurements in amateur soccer players* revealed mean depression values for the ADS in this study with 14.93 (SD=6.84). Compared with the ADS scale for men without lies this resulted in a per cent range of 68. A 13.47% of the amateur soccer players have a cut-off value of 23 or higher, which is a sign for a depression. The mean anxiety value for the STAI trait in this study was 37.39 (SD=9.64). Compared with the general male population aged between 15 and 29 years this equals a per cent range of 74. A 16.9% of the soccer players had a value of 45 or higher, which indicates an anxiety disorder. The mean value for self-compassion was 3.30 (SD=0.513).

Additionally, we found no main effect regarding the factor league for depression measurement (*F*(3,294)=1.96, p=0.120, η^2^=0.02) in this study. Thus, there was a main effect for the factor league for the anxiety measurement (*F*(3,292)=3.64, p=0.013, η^2^=0.036). Soccer players in the fourth amateur league showed a significant higher anxiety score than players in the fifth, sixth and seventh amateur leagues. There was one main effect for the factor league for the self-compassion measurement (*F*(3,295)=3.818, p=0.01, η^2^=0.037). Players of the sixth league showed a lower value compared to the players of the seventh league.

*The relation of psychological states and previous injuries* indicated varying results. There was no main effect for the factor league for the depression measurement (*F*(3,288)=1.794, p=0.149, η^2^=0.018). We also did not find a main effect for the factor group (*F*(1,288)=2.69, p=0.102, η^2^=0.009) and neither an interaction between both factors (*F*(3,288)=0.822, p=0.482, η^2^=0.008). There was one main effect for the factor league for the anxiety measurement (*F*(3,286)=3.39, p=0.019, η^2^=0.034) and one main effect for the factor group (*F*(1,286)=4.95, p=0.027, η^2^=0.017) but no interaction between both factors (*F*(3,286)=1.32, p=0.269, η^2^=0.014). As stated above, players from the fourth amateur league showed a higher anxiety score than players of the other three amateur leagues. Additionally, the anxiety score was higher for those players who suffered from an injury in the last 3 months before the season started (M=38.28, SD=10.82) compared with those who did not suffer from an injury during this time (M=36.06, SD=8.59). There was one main effect for the factor league for the self-compassion measurement (*F*(3,289)=3.070, p=0.028, η^2^=0.031). We did not find a main effect for the factor group (*F*(1,289)=2.791, p=p=0.096, η^2=^0.010) and neither an interaction between both factors (*F*(3,289)==0.662, p=0.576, η^2^=0.007).

There was no significant correlation between the frequency of injuries during the season and the psychological measurements at the beginning of the season (all p>0.4). However, all psychological measurements were highly significantly correlated (all p<0.001) (depression with anxiety, r=0.703 and self-compassion, r=−0.307; anxiety with self-compassion, r=−0.545).

## Discussion

The most important finding of our study is the frequency of basic anxiety in amateur soccer players. Our data concerning the psychological symptoms of amateur soccer players are partly in accordance with the data of Junge and Feddermann-Demont [5] in professional soccer showing almost exactly the same frequency of symptoms of anxiety.

### Psychological symptoms in amateur soccer players

The depression rate was slightly higher in our study of amateur players than the one in the study mentioned above in professional soccer. In addition to this, we could show that the anxiety value is higher for those soccer players who play in the highest league of the four amateur leagues investigated. Thus, we might conclude that if the performance pressure gets higher the anxiety rate increases. Even Junge and Feddermann-Demont [5] stated that a percentage of 16.7% is lower than the healthy population. Our result showed that almost 75% indicated a higher trait anxiety value than norm values of a non-athlete population at the same age range. We might conclude tentatively that if the professionalism gets higher in their careers anxiety does play a role, even though in this study we only investigated the trait component of anxiety and not the state one. The state anxiety component seemed to be more relevant to investigate the phenomenon of choking under pressure.^17^

Self-compassion as a positive component of emotional states did vary dependent on the league, but there was only one small effect between the players of the sixth and seventh league. However the values were significantly higher than the ones in a non-athlete US population of the same age.^18^ Even this result has to be taken with caution because of cross-comparison in two different populations, it gives a hint that research should focus not only the negative aspects of emotional states in soccer players but also on the positive ones. It might be possible that the highly trained athletes show a better self-compassion or a higher self-value than non-athletes and that this relates to other factors. This is in line with a study of Frost and McKelvie on 127 male and female students from elementary to high school and university.^19^ They found that high exercisers showed higher self-esteem regardless of exercise activity and that their self-esteem was robust across gender and age for all participants.

### Relation of injuries and psychological symptoms

Concerning the relation of injuries and psychological symptoms the results of our study demonstrated that an injury in the last 3 months before the measurement of the psychological symptoms leads to higher anxiety values. This result shows the need for further research and attention of psychological aspects after injuries. However, there was no relation in our study between the psychological states and the later injuries in the subsequent season. This result in amateur soccer is in line with a recently published study of Kiliç *et al*^8^ investigating national players of professional soccer. Both results show that the relation of psychological symptoms and injury is worth to be investigated in more depth. Injuries can be considered as stated by Kiliç *et al*^9^ as adverse life events in the life of soccer players and so they are likely to develop some negative psychological symptoms.

### Applied implications of the results

According to the study of Kiliç *et al*^8^ with professional soccer players this study indicates that injury can affect aspects of mental health even in non-elite soccer players. For this, an interdisciplinary medical approach in the treatment of injuries is needed in professional soccer players^8^ and in non-professional ones. This postulation is especially relevant for those soccer players who are at risk of mental health difficulties. An early identification of those players allows the prevention, before psychological symptoms during an injury get manifested. One might assume that this leads to a faster recovery and a more stressless season, because anxiety to injure is reduced.

### Strengths and limitations

An important strength of this study is the high number of amateur soccer players who participated in answering validated psychological tests. The compliance rate was 27.4% which is only slightly under the compliance rate of 34% in the study of Gouttebarge *et al*^7^ and which is more or less typical for soccer surveys with this topic, even we have to be aware that 72.6% did not respond. Another strength is the longitudinal character of this study. The longitudinal character allows the investigation of a possible causal relationship. However, besides those positive aspects there are some limitations: one of them is that the validated psychological measurements were retrieved in an online version, which the soccer players had to complete on their own; the other one is that the frequencies of injuries 3 months before the measurement were self-reported.

## Conclusion

In accordance with the study of Kiliç *et al*^8 9^ in professional soccer players, this study emphasises the importance of the attention to psychological factors also in lower soccer league levels, which might be related to injuries. Also amateur soccer players in higher leagues who suffer from injuries will develop some psychological concerns. We conclude that it is necessary to conduct more relevant studies investigating the possible relation of psychological and medical issues in soccer players.

## References

[1] Dvorak J, Junge A, Graf-Baumann T, et al Football is the most popular sport worldwide. Am J Sports Med 2004;32(1 Suppl):3S–4. 10.1177/0363546503262283 14754853

[2] Loose O, Fellner B, Lehmann J, et al Injury incidence in semi-professional football claims for increased need of injury prevention in elite junior football. Knee Surg Sports Traumatol Arthrosc 2019;27:978–84. 10.1007/s00167-018-5119-8 30167753

[3] Friedrich B, Mason OJ "What is the score?" A review of football-based public mental health interventions. J Public Ment Health 2017;16:144–58. 10.1108/JPMH-03-2017-0011 29721035 PMC5868541

[4] Chekroud SR, Gueorguieva R, Zheutlin AB, et al Association between physical exercise and mental health in 1·2 million individuals in the USA between 2011 and 2015: a cross-sectional study. Lancet Psychiatry 2018;5:739–46. 10.1016/S2215-0366(18)30227-X 30099000

[5] Junge A, Feddermann-Demont N Prevalence of depression and anxiety in top-level male and female football players. BMJ Open Sport Exerc Med 2016;2:e000087 10.1136/bmjsem-2015-000087 PMC5117057 27900164

[6] Gouttebarge V, Aoki H, Kerkhoffs G Symptoms of common mental disorders and adverse health behaviours in male professional soccer players. J Hum Kinet 2015;49:277–86. 10.1515/hukin-2015-0130 26925182 PMC4723178

[7] Gouttebarge V, Aoki H, Ekstrand J, et al Are severe musculoskeletal injuries associated with symptoms of common mental disorders among male European professional footballers? Knee Surg Sports Traumatol Arthrosc 2016;24:3934–42. 10.1007/s00167-015-3729-y 26233596 PMC5131082

[8] Kiliç Ö, Aoki H, Goedhart E, et al Severe musculoskeletal time-loss injuries and symptoms of common mental disorders in professional soccer: a longitudinal analysis of 12-month follow-up data. Knee Surg Sports Traumatol Arthrosc 2018;26:946–54. 10.1007/s00167-017-4644-1 28698928 PMC5847204

[9] Kiliç Ö, Aoki H, Haagensen R, et al Symptoms of common mental disorders and related stressors in Danish professional football and handball. Eur J Sport Sci 2017;17:1328–34. 10.1080/17461391.2017.1381768 28961069

[10] Sanders G, Stevinson C Associations between retirement reasons, chronic pain, athletic identity, and depressive symptoms among former professional footballers. Eur J Sport Sci 2017;17:1311–8. 10.1080/17461391.2017.1371795 28911275

[11] Neff KD The development and validation of a scale to measure Self-Compassion. Self and Identity 2003;2:223–50. 10.1080/15298860309027

[12] Leary MR, Hoyle RH, Handbook of individual differences in social behavior. New York, NY: Guilford Press, 2009.

[13] Mosewich AD, Crocker P RE, Kowalski KC, et al Applying self-compassion in sport: an intervention with women athletes. J Sport Exerc Psychol 2013;35:514–24. 10.1123/jsep.35.5.514 24197719

[14] Hautzinger M, Bailer M Allgemeine Depressions Skala: German Version of G-ADS. Göttingen: Beltz Test Verlag, 1993.

[15] Spielberger CD, Gorsuch RL, Lushene R, et al Manual for the State-Trait Anxiety Inventory. Palo Alto: Consulting Psychologists Press, 1983.

[16] Fuller CW, Ekstrand J, Junge A, et al Consensus statement on injury definitions and data collection procedures in studies of football (soccer) injuries. Br J Sports Med 2006;40:193–201. 10.1136/bjsm.2005.025270 16505073 PMC2491990

[17] Beilock S Choke: What the secrets of the brain reveal about getting it right when you have to. New York: ATRIA paperback, 2010.

[18] Raes F, Pommier E, Neff KD, et al Construction and factorial validation of a short form of the Self-Compassion scale. Clin Psychol Psychother 2011;18:250–5. 10.1002/cpp.702 21584907

[19] Frost J, McKelvie S The relationship of self-esteem and body satisfaction to exercise activity for male and female elementary school, high school, and university students. The Online Journal of Sport Psychology 2005;7:36–49.

